# A molecularly defined mPFC-BLA circuit specifically regulates social novelty preference

**DOI:** 10.1126/sciadv.adt9008

**Published:** 2025-04-23

**Authors:** Yiqiong Liu, Ying Wang, Guoguang Xie, Qianying Yang, Aritra Bhattacherjee, Chao Zhang, Yi Zhang

**Affiliations:** ^1^ Howard Hughes Medical Institute, Boston Children’s Hospital, Boston, MA 02115, USA.; ^2^ Program in Cellular and Molecular Medicine, Boston Children’s Hospital, Boston, MA 02115, USA.; ^3^ Division of Hematology/Oncology, Department of Pediatrics, Boston Children’s Hospital, Boston, MA 02115, USA.; ^4^ Department of Genetics, Harvard Medical School, Boston, MA 02115, USA.; ^5^ Harvard Stem Cell Institute, Boston, MA 02115, USA.

## Abstract

Social novelty preference is an important aspect of social interaction for evaluating new threats and opportunities for survival, but the underlying neuronal mechanism remains unclear. Here, we identify a molecularly defined medial prefrontal cortex (mPFC) excitatory neuron subtype, located in layer 5 expressing *Il1rapl2*, which is highly associated with social deficit disorders in genome-wide association studies and might be responsible for regulating social novelty preference. Using an *Il1rapl2*-Cre mouse line, we show that chemogenetic activation of the mPFC *Il1rapl2*-expressing neurons impairs social novelty preference but with little effect on sociability. In addition, fiber photometry recording indicates that this neuron subtype is inhibited when mice interact with novel but not with familiar mice. Furthermore, viral tracing and terminal manipulation reveal that basolateral amygdala (BLA)–projecting *Il1rapl2*^+^ neurons mediate the social novelty preference. Thus, our study uncovers a molecularly defined mPFC-BLA circuit that specifically regulates social novelty preference, highlighting that specific neuron subtypes and circuits could modulate distinct aspects of social behaviors.

## Teaser

BLA-projecting mPFC *Il1rapl2*^+^ neurons mediate the social novelty preference.

## INTRODUCTION

Social behaviors are innate, highly conserved motivational behaviors in humans and animals, with precise spatiotemporal choreographies of neural processes and coordination of oscillations within and between brains (*1*, *2*). Normal social behaviors are important for survival and reproduction in the ever-changing environment but are commonly disrupted in neurodevelopmental and neuropsychiatric disorders, such as autism and schizophrenia (*3*–*5*). Social novelty preference, the ability to recognize information that is incongruous with previous experience and tend to interact with one conspecific over another, is a trait displayed by gregarious animals and is critical for evaluating new threats, as well as opportunities for socializing or mating (*6*). Besides innate factors such as interacting with skin or opposite sex, social preference could be influenced by attention, perception, social memory, and hierarchy (*7*–*9*). However, the dynamics of behavioral sequences and neuronal circuits responsible for regulating social novelty preference are less understood.

The medial prefrontal cortex (mPFC) plays important roles in responding to social stimuli, decision-making, and cognition (*10*–*13*). Clinical and preclinical researches indicate that impaired social behavior is associated with structural, neurochemical, and excitatory alterations within the mPFC (*14*, *15*). Altered connectivity or activity of the mPFC has also been implicated in social deficits in individuals with autism (*16*, *17*), as well as in autism-like mouse models (*18*). Impaired function of excitatory neural ensembles in the mPFC has been associated with abnormal social exploration (*19*), while inhibition of γ-aminobutyric acid–releasing (GABAergic) neurons in the prelimbic (PrL) part of the mPFC disrupts social novelty behaviors (*20*). Notably, different mPFC neuron subtypes exhibit highly diverse roles in various social contexts (*21*–*23*). Recent studies have revealed a subpopulation of thin-tufted dopamine D1 receptor–expressing and nucleus accumbens (NAc)–projecting pyramidal neurons in the mouse mPFC involved in social memory (*24*). Moreover, an oxytocin receptor–expressing neuron cluster in PFC has been shown to specifically modulate social recognition (*25*). However, given the high heterogeneity of PFC neurons, whether there are molecularly identified neuron subtypes that specifically regulate social novelty preference is not clear.

In this study, we focus on the social novelty preference during social interaction and attempt to identify neural subtypes in the mPFC and the corresponding circuits that control social novelty. To this end, we integrated the transcriptomes of mPFC neuron subtypes (*26*, *27*) with the genome-wide association study (GWAS) datasets of various psychiatric diseases and identified an mPFC layer 5 (L5) neuron subtype that expresses interleukin-1 receptor accessory protein-like 2 (*Il1rapl2*), which is highly associated with social deficit disorders. To understand how this neuron subtype contributes to social deficit disorders, we generated an *Il1rapl2*-Cre mouse line. Chemogenetic activation of mPFC *Il1rapl2^+^* neurons impaired social novelty preference but did not affect sociability. In addition, Ca^2+^ recording indicated that mPFC *Il1rapl2^+^* neuron activity is inhibited when mice start interacting with novel mice but not with familiar mice. Furthermore, viral tracing and terminal manipulation reveal that a subset of BLA glutamatergic neurons mediates the mPFC *Il1rapl2^+^* neuron function in social novelty preference. Together, our study uncovers a molecularly defined mPFC–basolateral amygdala (BLA) circuit that specifically modulates social novelty preference, providing a potential target for social deficit treatment.

## RESULTS

### *Il1rapl2^+^* neurons represent an L5 glutamatergic neuron subtype in mPFC

**Fig. 1.**
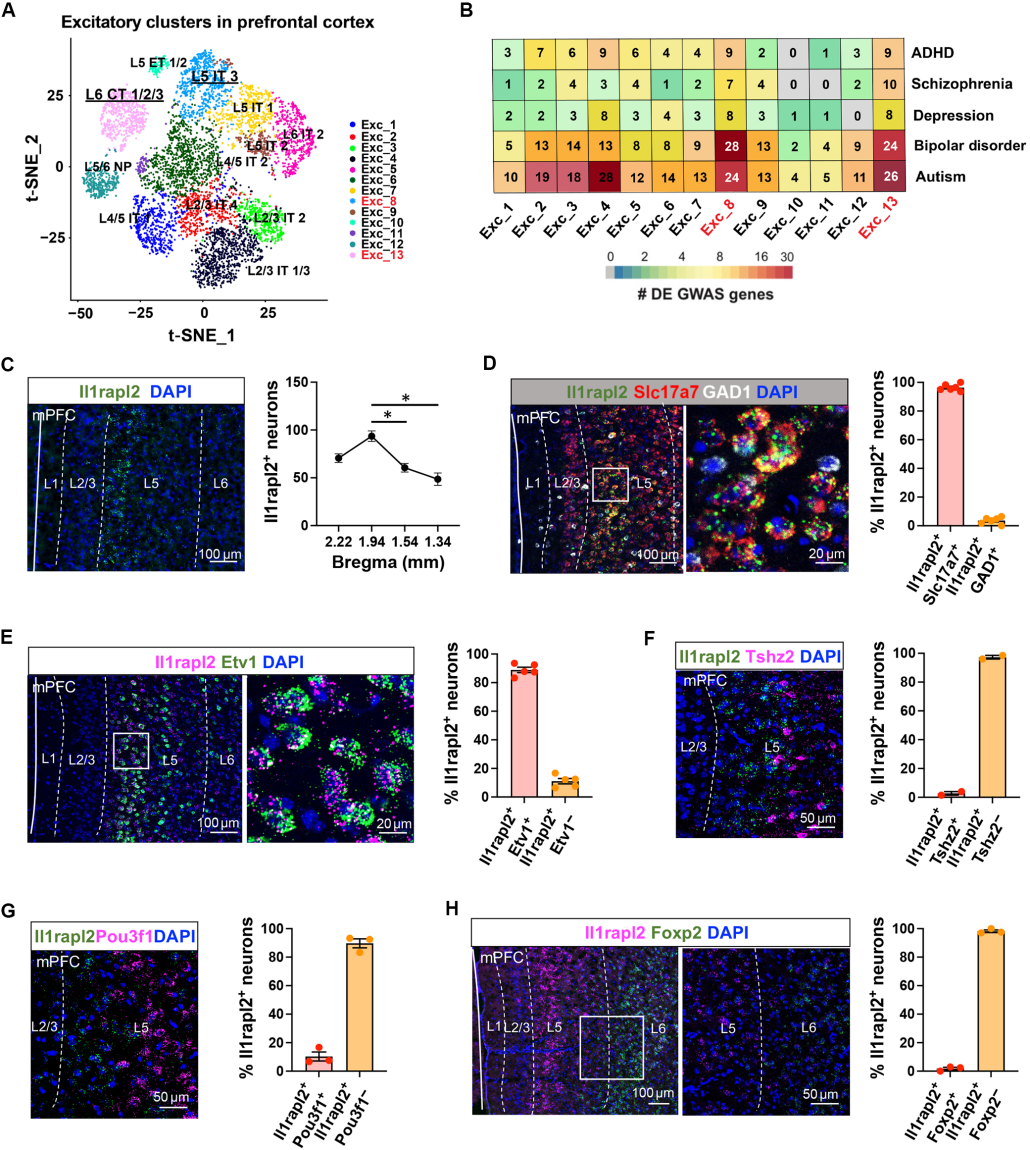
*Il1rapl2* marks an mPFC L5 IT neuron subtype. (**A**) t-SNE plot showing the 13 excitatory neuron subtypes of the mPFC classified on the basis of the transcriptome. NP, Near-projecting; t-SEN, t-distributed stochastic neighbor embedding. (**B**) Heatmap showing the number of differentially expressed GWAS candidate genes relevant to the five social-related psychiatric diseases in the mPFC excitatory neuron subtypes. ADHD, attention deficit hyperactivity disorder; DE, differentially expressed. (**C**) RNA in situ hybridization showing *Il1rapl2* mRNA expression in the mPFC (left). The average number of *Il1rapl2*-expressing neurons in the mPFC from rostral to caudal sections (right). *P < 0.05; one-way analysis of variance (ANOVA) and post hoc test. Scale bar, 100 μm. *n* = 3 mice. (**D**) Coexpression of *Il1rapl2* mRNA with *Slc17a7* (excitatory neuron marker) and *GAD1* (inhibitory neuron marker) mRNA in the mPFC. The boxed region is enlarged. The percentages of *Il1rapl2*-expressing neurons overlapped with *Slc17a7* and *GAD1* are shown on the right. *n* = 6 mice. (**E**) Coexpression of *Il1rapl2* mRNA with *Etv1* (L5 marker) mRNA in the mPFC. The boxed region is enlarged. The percentage of *Il1rapl2*-expressing neurons overlapped with *Etv1* is shown on the right. *n* = 5 mice. (**F**) Coexpression of *Il1rapl2* mRNA with *Tshz2* (an L5 cluster marker) mRNA in the mPFC. The percentage of *Il1rapl2*-expressing neurons overlapped with *Tshz2* is shown on the right. *n* = 2 mice. (**G**) Coexpression of *Il1rapl2* mRNA with *Pou3f1* (an L5 cluster marker) mRNA in the mPFC. The percentage of *Il1rapl2*-expressing neurons overlapped with *Pou3f1* is shown on the right. *n* = 3 mice. (**H**) Coexpression of *Il1rapl2* mRNA with *Foxp2* (an L6 marker) mRNA in the mPFC. The boxed region is enlarged. The percentage of *Il1rapl2*-expressing neurons overlapped with *Foxp2* is shown on the right. *n* = 3 mice.

Although the mPFC has long been implicated in encoding social behavior, given the tremendous neuron heterogeneity of this brain region, it is not clear whether different neuron subtypes of the mPFC can encode different aspects of social behavior, such as sociability and social novelty preference. By performing single-cell spatial transcriptomic analysis, we previously classified the mouse mPFC excitatory neuron into 13 major subtypes (Ext_1 to Exc_13) that include intratelencephalic (IT), extratelencephalic (ET), and corticothalamic (CT) neurons with their layer information (*28*) (Fig. 1A). To identify which of the 13 neuron subtypes might be involved in a particular psychiatric disease, we calculated the number of differentially expressed GWAS candidate genes by comparing the single-cell RNA sequencing (scRNA-seq) data in postnatal day 21 (p21) and p60 mPFC in each of the 13 neuron subtypes (*27*). This analysis identified Exc_8 and Exc_13 as the neuron subtypes having the most number of differentially expressed GWAS genes associated with autism and bipolar disorder (Fig. 1B), two syndromes with social defect as a hallmark of the diseases (*5*, *29*, *30*). The expression of *Il1rapl2* or *forkhead box p2* (*Foxp2*) is respectively enriched in these two neuron subtypes (fig. S1, A and B).

Our scRNA-seq results indicate that *Foxp2*-expressing neurons belong to layer 6 (L6) CT projection neurons (Fig. 1A and fig. S1B), consistent with previous results (*31*–*33*). To determine the spatial distribution of the *Il1rapl2^+^* neurons, we performed single-molecule fluorescence in situ hybridization (smFISH), which revealed that the *Il1rapl2* signal can be detected along the anterior-posterior (AP) axis but with the great majority of signals detected in L5, which peaks at bregma +1.94 (Fig. 1C). Costaining *Il1rapl2* with *Slc17a7* (solute carrier family 17 member 7, glutamatergic neuron marker) and *GAD1* (glutamate decarboxylase 1, GABAergic neuron marker) revealed that about 98% *Il1rapl2^+^* neurons are glutamatergic neurons (Fig. 1D). In addition, about 90% of *Il1rapl2^+^* neurons overlap with the L5 marker *ETS variant transcription factor 1* (*Etv1*) (Fig. 1E). Because *teashirt zinc finger homeobox 2* (*Tshz2*) and *POU class 3 homeobox 1* (*Pou3f1*) have also been reported to mark mPFC L5 neuron subtypes (*27*), we asked whether *Il1rapl2* colocalizes with *Tshz2* or *Pou3f1*. smFISH revealed that there is little colocalization between *Il1rapl2* and *Tshz2* or *Pou3f1* (Fig. 1, F and G). Furthermore, there is almost no overlap between *Il1rapl2* and the L6 CT neuron marker *Foxp2* (Fig. 1H). Collectively, these results indicate that *Il1rapl2-*expressing neurons represent a previously uncharacterized L5 glutamatergic neuron subtype in the mPFC. Because both the *Il1rapl2^+^* L5 IT neurons and the *Foxp2^+^* L6 CT neurons in the mPFC have the most number of differentially expressed GWAS genes for bipolar disorder and autism, they might be involved in regulating aspects of social behaviors.

### Activation of the mPFC *Il1rapl2^+^* neurons disrupts social novelty preference

To determine whether the mPFC *Il1rapl2^+^* L5 neurons and the *Foxp2^+^* L6 neurons are involved in social behavior, we performed the three-chamber social interaction test in mice with or without manipulation of the neuronal activity of these two neuron subtypes. To this end, Cre-dependent adeno-associated viruses (AAVs) expressing AAV-DIO-hM3Dq-mCherry or AAV-DIO-hM4Di-mCherry were injected into the mPFC L6 of the *Foxp2*-Cre mice with the AAV-DIO-mCherry serving as a negative control (fig. S2, A and B). Then, clozapine *N*-oxide (CNO) was intraperitoneally injected to activate or inhibit mPFC *Foxp2^+^* neurons 30 min before the social behavior test. During the classic three-chamber social interaction test, the mice experienced 10 min of the habituation phase, 10 min of the sociability phase, and 10 min of the social novelty preference phase (fig. S2C, top), and social novelty preference is measured by a shift of interest from a familiar social stimulus to a novel one when both are present (*34*). The results indicate that neither activation nor inhibition of the mPFC *Foxp2^+^* neurons affected social behaviors (fig. S2, C and D), indicating that the mPFC *Foxp2^+^* neurons are not involved in social behavior. Thus, we turned our attention to the mPFC L5 *Il1rapl2^+^* neuron subtype.

**Fig. 2.**
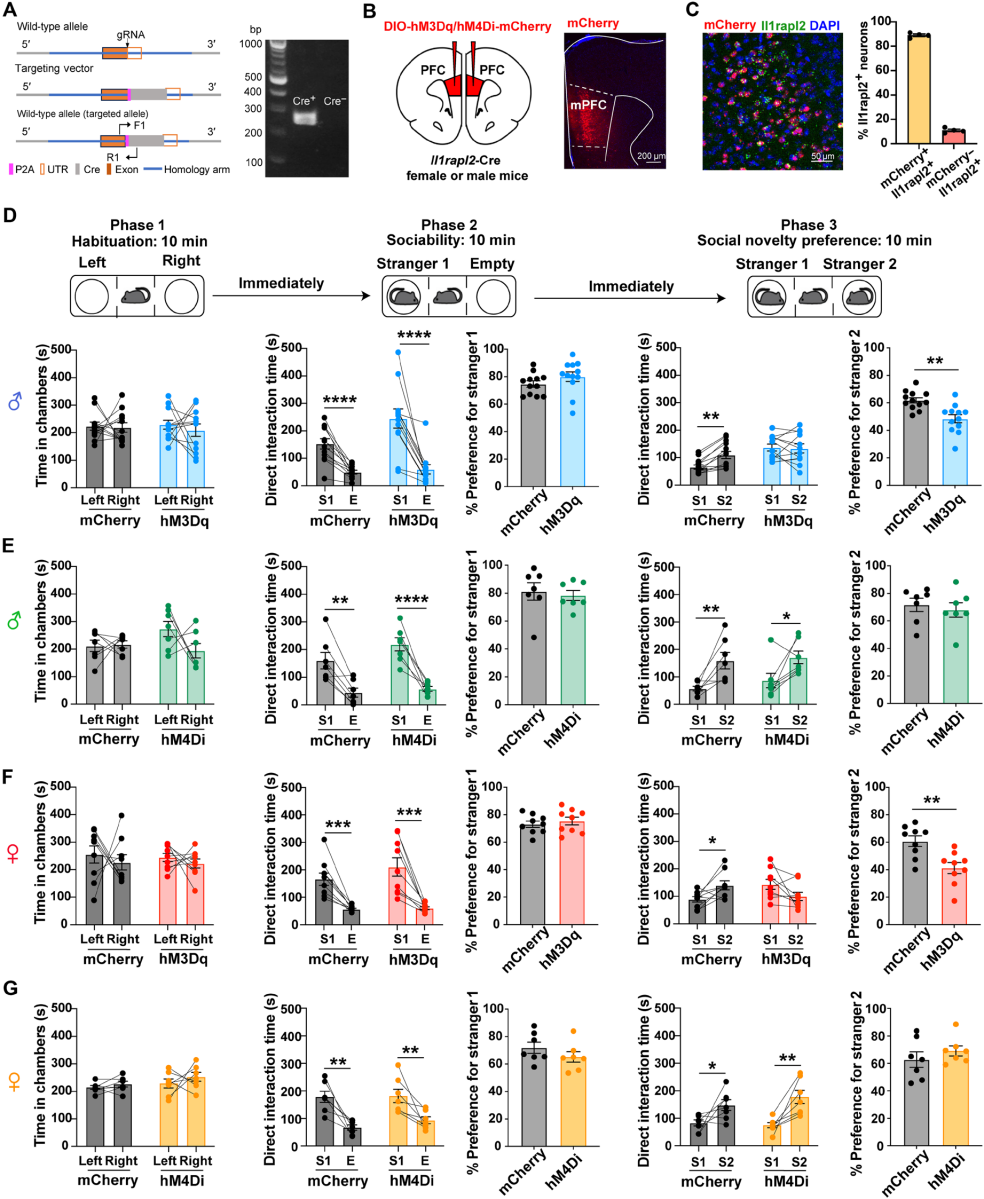
Hyperactivation of mPFC *Il1rapl2^+^* neurons disrupts social novelty preference. (**A**) Diagrams showing the gene targeting strategy (left) to generate the *Il1rapl2*-Cre mouse line and genotyping by polymerase chain reaction (PCR; right). Cre*^+^*: 252 base pairs (bp). gRNA, guide RNA; P2A, porcine teschovirus-1 2A; UTR, untranslated region; F1, forward primer 1; R1, reverse primer 1. (**B**) Diagram showing the injection site of AAV-DIO-hM3Dq/hM4Di-mCherry virus in the mPFC of *Il1rapl2*-Cre mice (left) and the confirmation of mCherry expression in the mPFC (right). (**C**) RNA in situ hybridization showing the overlap between mCherry and *Il1rapl2* mRNA (left) and the quantification of the overlap (right). *n* = 4 mice. (**D**) Three-chamber interaction test on male *Il1rapl2*-Cre mice in both mCherry and hM3Dq groups in the habituation phase (left), the sociability phase (middle), and the social novelty preference phase (right). **P < 0.01 and ****P < 0.0001; Student’s *t* test. E, empty; S1, stranger 1; S2, stranger 2. (**E**) Three-chamber interaction test on male *Il1rapl2*-Cre mice in both mCherry and hM4Di groups in the habituation phase (left), the sociability phase (middle), and the social novelty preference phase (right). *P < 0.05, **P < 0.01, and ****P < 0.0001; Student’s *t* test. (**F**) The same as (D) except that the test was performed using female mice. *P < 0.05, **P < 0.01, and ***P < 0.0001; Student’s *t* test. (**G**) The same as (F) except that the test was performed using female mice. *P < 0.05 and **P < 0.01; Student’s *t* test.

To determine whether the mPFC L5 *Il1rapl2^+^* neurons have a role in social behaviors, we first generated an *Il1rapl2*-Cre transgenic mouse line, which was confirmed by allele-specific genotyping (Fig. 2A). To determine whether mPFC *Il1rapl2^+^* neurons play a causal role in regulating social behavior, we bilaterally injected the chemogenetic activation viruses AAV-DIO-hM3Dq-mCherry or the inhibitory viruses AAV-DIO-hM4Di-mCherry into the mPFC with the AAV-DIO-mCherry serving as a control (Fig. 2B). To confirm the expression of mCherry in the *Il1rapl2^+^* neurons, we performed smFISH and found about 90% of *Il1rapl2*^+^ neurons expressed mCherry in the mPFC (Fig. 2C). The three-chamber social interaction behavioral test indicated that chemogenetic activation of mPFC *Il1rapl2^+^* neurons in male mice disrupted social novelty preference but has little effect on sociability (Fig. 2D). However, chemogenetic inhibition of the mPFC *Il1rapl2^+^* neurons affected neither social novelty preference nor sociability (Fig. 2E). Neither chemogenetic activation nor inhibition affected locomotion behaviors (fig. S3). Because social behavior exhibits gender bias (*35*, *36*), we repeated the test in female mice and observed a similar result (Fig. 2, F and G), indicating no sex bias of mPFC *Il1rapl2^+^* neurons in social novelty regulation. Collectively, these results indicate that the activation of the mPFC *Il1rapl2^+^* neurons is sufficient to disrupt social novelty preference in both male and female mice.

Because social novelty preference reflects social memory, we next asked whether mPFC *Il1rapl2^+^* neurons also regulate other types of memory, such as working memory or associative learning memory. To this end, we performed novel object recognition and cued fear conditioning memory tests and found that the chemogenetic activation of mPFC *Il1rapl2^+^* neurons does not affect object novelty memory or cued fear conditioning memory (fig. S4). Together, these results indicate that mPFC *Il1rapl2^+^* neurons specifically regulate social novelty memory, but not working memory or associative learning memory.

### The *Il1rapl2^+^* neuron activity decreases when mice interact with novel mice

**Fig. 3.**
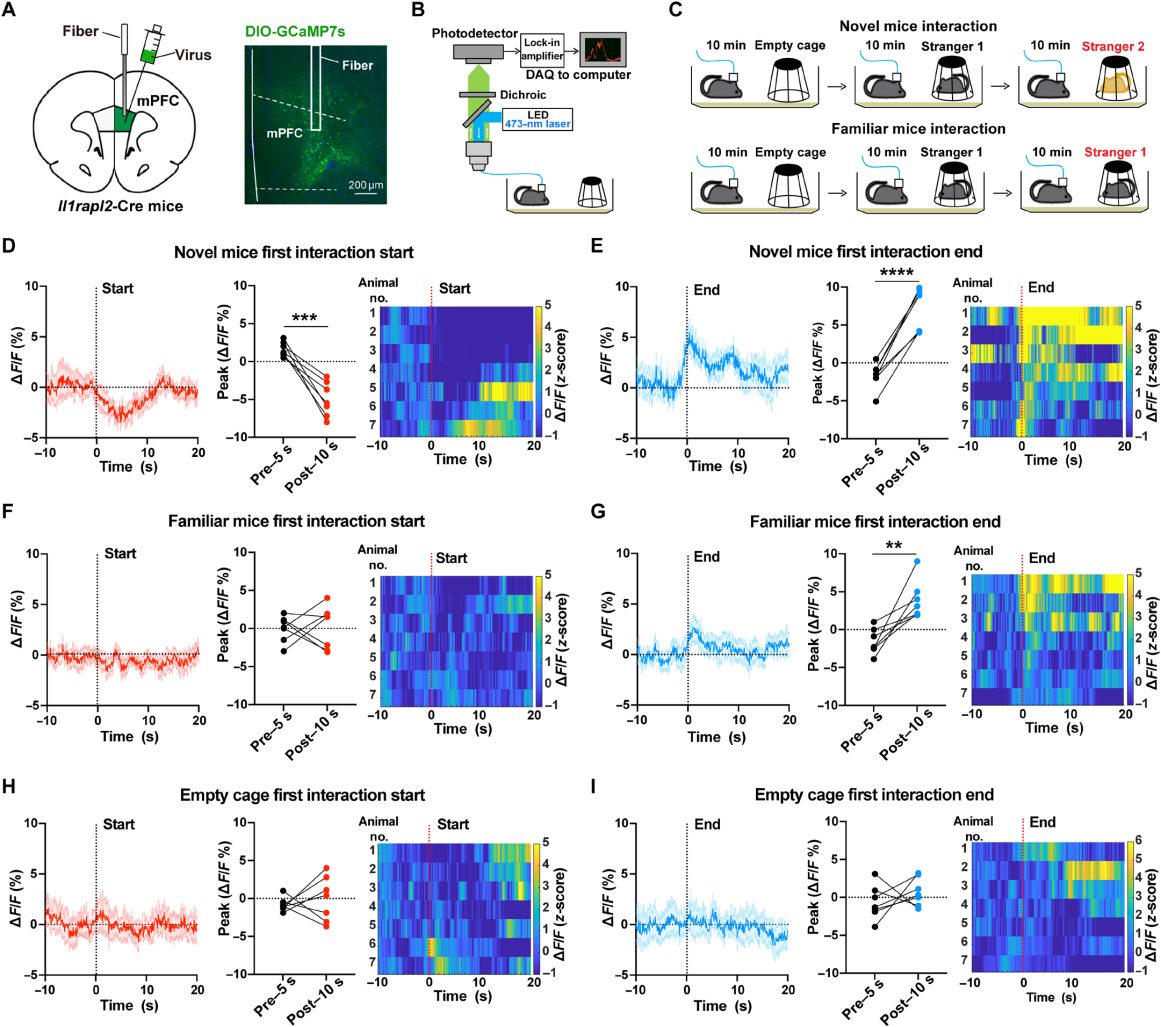
mPFC *Il1rapl2^+^* neurons respond to social behavior. (**A**) Diagram of the injection site of AAV-DIO-GCaMP7s virus in the mPFC of *Il1rapl2*-Cre mice (left) and the confirmation of GCaMP7s expression in the mPFC (right). (**B**) Diagram for fiber photometry. LED, light-emitting diode; DAQ, data acquisition. (**C**) Schematic setup for monitoring mPFC *Il1rapl2^+^* neuronal activity when facing familiar and novel mice. (**D** to **I**) Averaged peristimulus traces (left), quantification of peak Δ*F/F* (in percentage) (middle), and corresponding heatmaps of Ca^2+^ signals of mPFC *Il1rapl2^+^* neurons at the start and end of social interactions with novel mice [(D) and (E)], familiar mice [(F) and (G)], and empty cages [(H) and (I)]. Animals were aligned on the basis of averaged *z*-score of post–10 s from lowest to highest for (D), (F), and (H) and from highest to lowest for (E), (G), and (I). **P < 0.01, ***P < 0.001, and ****P < 0.0001; paired *t* test.

To determine the dynamics of mPFC L5 *Il1rapl2^+^* neuronal activity in social interaction especially when interacting with familiar or novel mice, we used fiber photometry to monitor the real-time neuronal activity changes. To this end, Cre-dependent AAVs encoding a calcium-activated green fluorescent protein GCaMP7s were delivered to the mPFC of *Il1rapl2*-Cre mice, and an optic fiber was implanted above the virus injection site (Fig. 3A). Three weeks after the surgeries, fluorescence recording during social interaction with familiar or novel mice in the home cage was performed (Fig. 3, B and C). The results indicate that the Ca2^+^ signals of the mPFC *Il1rapl2^+^* neurons decreased immediately when the mice started to interact with novel mice (Fig. 3D), but not with familiar mice or an empty cup (Fig. 3, F and H). To avoid neuronal adaptation after several times of interaction with mice, we only used the first-time interaction for analysis (*37*). To quantify the *Il1rapl2^+^* neuronal activity at different events, we averaged the calcium signals of each animal and observed that the peak average signals between the pre- and postevent period are significantly decreased after the interaction with the novel mice starts (Fig. 3D), while the activity of the mPFC *Il1rapl2^+^* neurons increased immediately when interaction with novel mice ends (Fig. 3E) but less increased with familiar mice or no significant change with an empty cage (Fig. 3, G and I). Collectively, these results indicate that *Il1rapl2^+^* neurons exhibit a special response to novel mice, but not to familiar mice or an empty cage, and that the neuronal activity is significantly decreased upon interacting with novel mice.

### mPFC *Il1rapl2^+^* neurons receive multiple inputs and mainly project to BLA and PT

**Fig. 4.**
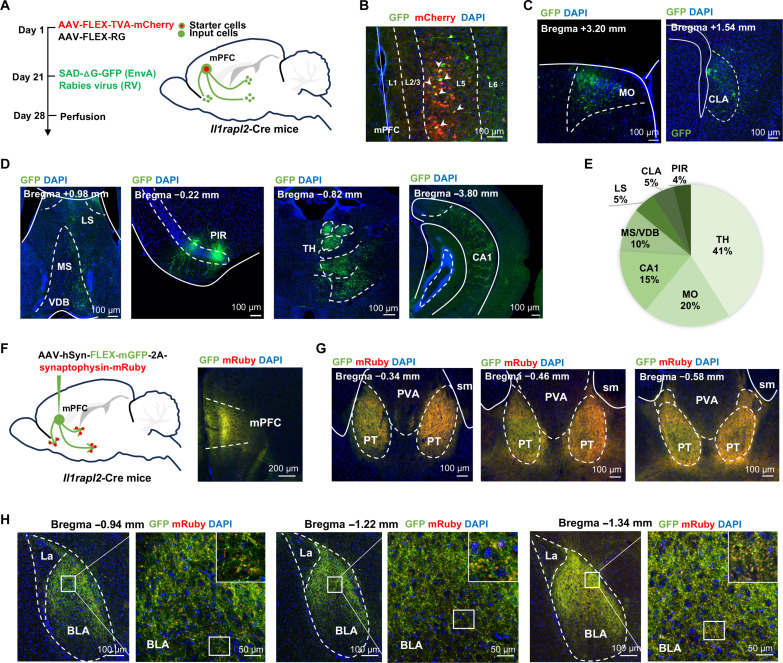
mPFC *Il1rapl2^+^* neurons receive multiple inputs and mainly project to BLA and PT. (**A**) Schematic diagram of modified RV–mediated retrograde monosynaptic tracing. (**B**) Representative image of the TVA-mCherry and EnvA-GFP double-labeled starter cells in the mPFC from an *Il1rapl2*-Cre mouse injected with retrograde tracing viruses. (**C** and **D**) Representative images of GFP^+^ presynaptic neurons in the primary motor cortex (MO), claustrum (CLA), medial septum (MS) or nucleus of the vertical limb of the diagonal band (VDB), piriform cortex (PIR), thalamus (TH), and field CA1 of the hippocampus (CA1). (**E**) Proportion of GFP*^+^* neurons in various brain regions with monosynaptic projection to the mPFC *Il1rapl2*^+^ neurons, relative to all retrograde-labeled cells (*n* = 3 brains). (**F**) Schematic diagram showing the strategy for viral labeling of mPFC *Il1rapl2*^+^ neurons for anterograde tracing with AAV-FLEX-mGFP-2A-synaptophysin-mRuby (left) and a representative image confirming virus expression (right). (**G** and **H**) Representative images showing the projection of mPFC *Il1rapl2^+^* neuron terminals with green fibers and red puncta in two downstream target brain areas, PT (G) and BLA (H). sm, stria medullaris; PVT, paraventricular nucleus of the thalamus; LA, lateral amygdalar nucleus.

To dissect the circuitry mechanism underlying mPFC *Il1rapl2^+^* neuron subtype–mediated social novelty preference regulation, we first attempted to identify the inputs of mPFC *Il1rapl2^+^* neurons. To this end, we performed monosynaptic retrograde tracing by injecting Cre-dependent AAVs expressing transmembrane viral adhesion protein (TVA) and rabies glycoprotein (RG) into the mPFC of the *Il1rapl2*-Cre mice, followed by microinjection of glycoprotein(G)-deleted rabies virus pseudotyped with EnvA (the avian sarcoma leucosis virus glycoprotein) expressing green fluorescent protein (RV-EnvA-ΔG-GFP) 3 weeks later (Fig. 4A). After confirming the virus expression (Fig. 4B), we mapped the upstream input neurons labeled with GFP across the entire brain 7 days later. We found multiple brain regions targeting the mPFC *Il1rapl2^+^* neurons, including primary motor cortex (MO), claustrum (CLA) (Fig. 4C), medial septum (MS) or vertical limb of diagonal band (VDB), piriform cortex (PIR), thalamus (TH), and the field CA1 of hippocampus (CA1) (Fig. 4D). Quantification indicated that TH is the major input for the mPFC *Il1rapl2^+^* neurons (Fig. 4E).

To determine the downstream projection sites of mPFC *Il1rapl2^+^* neurons, we injected Cre-dependent AAVs expressing membrane-bound GFP (mGFP; for axon labeling) and synaptophysin-mRuby (for presynaptic site labeling) into the mPFC of the *Il1rapl2*-Cre mice (Fig. 4F). Four weeks later, we observed abundant green neuronal terminals and red presynaptic puncta in paratenial thalamic nucleus (PT) and BLA (Fig. 4, G and H) but not in dorsal striatum, NAc, hippocampus, periaqueductal gray, or ventral tegmental area (fig. S5). Collectively, these results indicate that the mPFC *Il1rapl2^+^* neurons receive inputs mainly from TH and send output terminals mainly to PT and BLA.

### *Il1rapl2^+^* neurons regulate social novelty preference via BLA projection

**Fig. 5.**
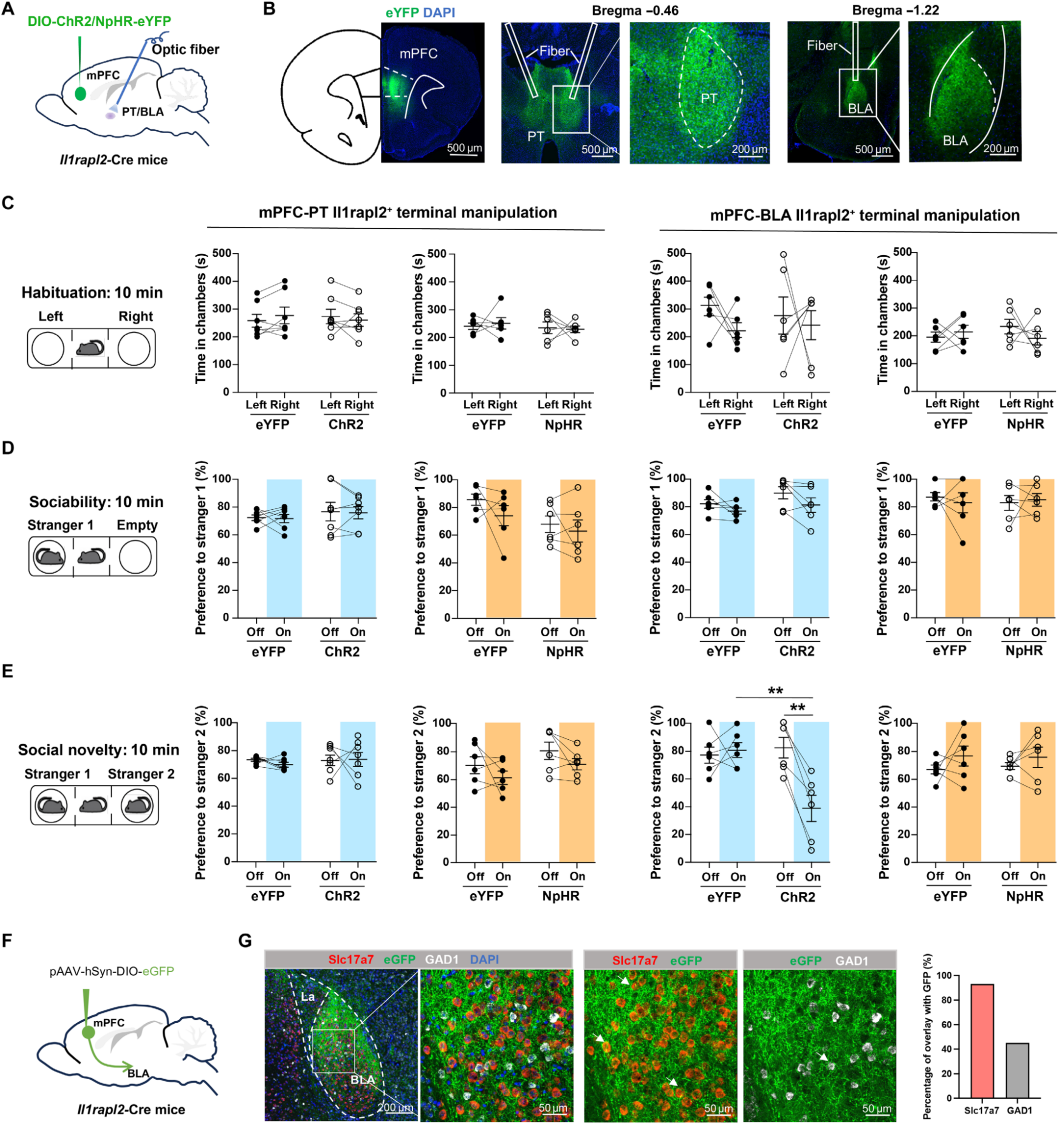
*Il1rapl2^+^* neurons regulate social novelty preference via BLA projection. (**A**) Schematic diagram showing the delivery of AAV into the mPFC and the optic cannula implantation in PT or BLA in *Il1rapl2*-Cre mice. (**B**) Representative images confirming virus expression in the mPFC and terminals in PT and BLA. (**C**) Time spent investigating left and right chambers during the habituation stage in eYFP, ChR2, and NpHR groups in *Il1rapl2*-Cre mice with manipulation of mPFC-PT (left) or mPFC-BLA (right) *Il1rapl2*^+^ terminals. (**D**) Preference percentage to stranger 1 during sociability stage in eYFP, ChR2, and NpHR groups in *Il1rapl2*-Cre mice with manipulation of mPFC-PT (left) or mPFC-BLA (right) *Il1rapl2*^+^ terminals. (**E**) Preference percentage to stranger 2 during sociability stage in eYFP, ChR2, and NpHR groups in *Il1rapl2*-Cre mice with manipulation of mPFC-PT (left) or mPFC-BLA (right) *Il1rapl2*^+^ terminals. **P < 0.01; Student’s *t* test. (**F**) Schematic diagram showing delivery of AAV-hSyn-DIO-eGFP virus in the mPFC in *Il1rapl2*-Cre mice. (**G**) Representative images and quantification of overlaps between mPFC *Il1rapl2*^+^ GFP terminals with *Slc17a7^+^* or *GAD1^+^* neurons in BLA.

Next, we attempted to investigate whether the PT or BLA projection of mPFC *Il1rapl2^+^* neurons is functionally important in mediating the social novelty preference by optogenetic manipulation of the mPFC *Il1rapl2^+^* neuronal terminals. Given that *Il1rapl2^+^* neurons exhibit no sex difference in regulating social interaction (Fig. 2), we used male mice for optogenetic manipulation for simplicity. AAV-DIO-ChR2-eYFP or halorhodopsin (AAV-DIO-eNpHR-eYFP) were injected into the mPFC of the *Il1rapl2*-Cre mice with optic fiber implanted into the PT or BLA while AAV-DIO-eYFP was used as a control (Fig. 5A). After confirming the eYFP expression in PT and BLA (Fig. 5B), the mice were subjected to the social behavior test. We found that optogenetic activation of the mPFC-BLA, but not the mPFC-PT, circuit only disrupted social novelty preference (Fig. 5, C to E, blue bars). However, optogenetic inhibition of mPFC-BLA *Il1rapl2^+^* projection did not significantly alter the social novelty preference (Fig. 5, C to E, yellow bars). Neither optogenetic activation nor inhibition affected locomotion behaviors (fig. S6). Moreover, when analyzing the immediate early gene -c-fos expression in the mPFC and BLA after novel mouse interaction (fig. S7A), we found that mPFC and BLA neurons are coordinately inhibited when approaching novel mice as indicated by the decreased c-Fos^+^ neuron numbers (fig. S7, B and C). To exclude a potential secondary anxiety effect of the PFC-BLA circuit on social choice, we performed the elevated O-maze to determine whether PFC-BLA circuit manipulation affects anxiety. We found that activation of the mPFC*^Il1rapl2^*-BLA circuit does not affect anxiety (fig. S8). Thus, we conclude that the mPFC *Il1rapl2^+^* to the BLA projection is responsible for the social novelty preference function of the mPFC *Il1rapl2^+^* neurons.

Then, we attempted to identify the downstream neuronal types in BLA, which receive innervation from mPFC *Il1rapl2^+^* neurons. BLA neurons can be broadly divided into *Slc17a7*– and *GAD1*–expressing neuron types (*38*). Therefore, we injected the AAV-hSyn-DIO-eGFP viruses to the mPFC of the *Il1rapl2*-Cre mice and performed smFISH for *Slc17a7* and *GAD1* on slices covering the BLA (Fig. 5F and fig. S9). We found that presynaptic terminals (green) of mPFC *Il1rapl2^+^* neurons are mainly located around the *Slc17a7^+^* neurons (Fig. 5G). The above result indicates that hyperactivation of mPFC *Il1rapl2^+^* neurons probably further activates BLA glutamatergic neurons, which impairs social novelty preference.

## DISCUSSION

Most primates, including humans, live in social groups where they interact with members of the group. The neuronal identity and circuit mechanisms underlying social novelty preference and group behavior are complex and remain to be elucidated. By integrating scRNA-seq with the differential expression of the GWAS genes of major psychiatric disorders in different excitatory neuron subtypes of p21 and p60 mPFCs, we identified an mPFC L5 IT neuron subtype, marked by the expression of the *Il1rapl2* gene, characterized by differential expression of a large number of genes associated with social deficit disorders such as autism and bipolar disorder (Fig. 1B, Exc_8). We showed that chemogenetic activation of mPFC *Il1rapl2^+^* neurons is sufficient to impair social novelty preference, but not sociability (Fig. 2, D to G). Moreover, this neuron subtype is specifically inhibited when interacting with novel mice, but not with familiar mice or an empty cage (Fig. 3, D, F, and H). Viral tracing and terminal manipulation further demonstrate that the mPFC *Il1rapl2*^+^ neurons to BLA projection, but not the mPFC to PT projection, mediates social novelty preference (Fig. 5, C to E). Thus, our study reveals a neural circuit underlying social novelty preference with brain region–, circuit-, and neuron subtype–specificity in mice, which provides direct evidence supporting the notion that different aspects of social behavior can be regulated by specific mPFC neuron subtypes and circuits.

### mPFC census in social behavior

The mPFC is important for social information processing (*12*, *13*, *39*), but the mechanisms by which mPFC neurons encode real-time social exploration remain largely unknown. Previous studies have shown that PrL neurons projecting to the NAc are responsible for processing social and spatial information during social interaction (*40*). Using Ca^2+^ imaging, people have also revealed distinct and dynamic on and off neural ensembles in the PFC that encode social exploration (*19*). However, the lack of genetic and molecular identity of these neural ensembles makes it difficult to target and recapture these neurons for further study. Thus, one important question is to identify the molecularly defined neuron subtypes in the highly heterogeneous mPFC neurons encoding the different aspects of social behaviors. Using single-cell spatial transcriptome techniques, we recently deciphered the neuronal heterogeneity of the mPFC and identified molecularly and spatially distinctive neuronal subtypes (*27*, *41*), which makes the identification of specific mPFC neuronal subtypes encoding different aspects of social behavior possible. By analyzing the expression dynamics of GWAS genes linked to different psychiatric diseases in the various mPFC neuron subtypes, we identified *Il1rapl2*- and *Fopx2*-labeled neuron subtypes that are highly enriched for differentially expressed genes in p21 and p60 mPFCs related to social deficit diseases (Fig. 1B). Only manipulating *Il1rapl2*^+^ neurons can specifically affect social novelty preference (Fig. 2 and fig. S2). It is likely that *Fopx2*^+^ neurons might be involved in modulating other autism- or bipolar disorder–related behaviors, which is worth further exploration. Our results support the notion that different neuron subtypes of the mPFC encode different behaviors that the mPFC regulates.

### mPFC-BLA circuit regulates social novelty preference

Previous studies have shown that the PFC-BLA circuit plays a central role in regulating social behavior (*42*–*44*) and abnormal social behaviors and that deficits in the PFC-BLA circuit were observed in several autism mouse models (*45*, *46*). Moreover, in response to a social cue, the infralimbic cortex (IL)–BLA projecting neurons are preferentially activated compared to PrL-BLA projecting neurons (*39*). The differential function of PrL- and IL-BLA circuitry in social behaviors likely arises from differences in connectivity in these subcircuits. In our study, optogenetic manipulation of the mPFC*^Il1rapl2^-*BLA circuit does not modulate sociability but social novelty (Fig. 5). We do not consider these results to be in conflict, as *Il1rapl2*^+^ neurons only represent 1 of the 13 excitatory neuron subtypes, a small fraction of the mPFC neurons (Fig. 1). Manipulation of the mPFC-BLA circuit could have different effects on social behavior compared to manipulation of the mPFC*^Il1rapl2^-*BLA circuit, as other neuron subtypes could also project to BLA. On the other hand, studies have shown that two types of neurons exist in the BLA: One type is activated by positive valence, and the other is activated by negative valence (*47*, *48*). Combined with recent spatial transcriptomic profiling of BLA (*49*), our study has identified the Slc17a7^+^ neurons as the major downstream target of mPFC *Il1rapl2^+^* neurons in BLA (Fig. 5). Different neuronal subtypes of BLA receiving mPFC input could also explain the different results. Whether different *Slc17a7*^+^ subtypes are involved in regulating different aspects of social behavior remains to be determined. Optogenetic manipulation of the mPFC*^Il1rapl2^-*BLA circuit in three-chamber social interaction leads to a decrease in novelty preference (Fig. 5). Whether the decrease is due to an interference in the encoding of social memory formation or retrial remains to be determined. Thus, identifying the specific neuron subtypes for a specific aspect of social behavior could allow us to better understand how different molecular and cellular mechanisms are integrated in the mPFC-to-BLA circuits to regulate different aspects of social behavior.

### Neuronal networks that regulate social novelty

Neurodevelopment disorders, such as autism, are associated with excitation-inhibition imbalance that alters the function of neural circuits controlling social behavior (*50*). A hallmark of autism is the difficulty in performing tasks that require face recognition, which is the ability to identify a person based on memory of facial features (*51*). In typical subject recognition, repeatedly viewing the same face is associated with decreased neural activity within subdivisions of the PFC (*52*), which is attenuated in people with autism (*15*). Our Ca^2+^ imaging result indicates that *Il1rapl2^+^* neuronal activity significantly decreases upon interacting with novel mice (Fig. 3), which is consistent with the previous study (*15*).

Different neuron subtypes in the mPFC work in concert to keep excitatory/inhibitory balance (*53*). Our study uncovers a specific mPFC excitatory neuronal subtype whose hyperactivation erases the social novelty preference. We believe that some other neuronal subtypes can function compensatorily to maintain the excitatory/inhibitory balance. In addition to the downstream BLA, we mapped the upstream regions of mPFC *Il1rapl2^+^* neurons to be mainly the thalamic area (Fig. 4). Given that TH is a social behavior–related region (*54*–*59*), we speculate that hyperexcitation of thalamic innervation to the mPFC can also contribute to mPFC-mediated disruption of social novelty preference, which is yet to be shown.

Collectively, our study identifies a molecularly defined top-down neural circuit that specifically encodes social novelty preference, but not sociability. Our data suggest that the hyperactivation of the mPFC *Il1rapl2^+^* neurons is sufficient to disrupt social novelty preference mediated by downstream BLA glutamatergic neurons, which provides notable information about the role of PFC-amygdala circuit in social behavior and a potential therapeutic target for autism treatment.

## MATERIALS AND METHODS

### Animals

All experiments were conducted in accordance with the National Institutes of Health *Guide for the Care and Use of Laboratory Animals* and approved by the Institutional Animal Care and Use Committee of Boston Children’s Hospital and Harvard Medical School. B6.Cg-Foxp2^tm1.1(cre)Rpa^/J (Jax:030541) and C57BL/6NJ (Jax:000664) mice were purchased from the Jackson Laboratory.

To generate the *Il1rapl2*-Cre mouse line, a selection cassette containing the porcine teschoviral 2A cleavage sequence linked to Cre recombinase was targeted to replace the stop codon of the *Il1rapl2* gene in a bacterial artificial chromosome. A targeting plasmid containing the Cre-containing selection cassette and ~4-kb genomic sequence upstream and downstream of the *il1rapl2* stop codon was isolated and used for embryonic stem cell targeting. Correctly targeted clones were identified by quantitative polymerase chain reaction (PCR) from embryonic stem cell clone DNA. Chimeric animals generated from blastocyst implantation were then bred for germline transmission of the *Il1rapl2*-Cre allele. Genotyping was performed using allele-specific PCR. Forward primer: 5′-CACACTTACTGTAACTTGCCACTG-3′. Reverse primer: 5′-TACGGTCAGTAAATTGGACACCTT-3′.

For behavioral assays, 12- to 16-week-old male or female mice were used. The mice were housed in groups (three to five mice per cage) in a 12-hour light/dark cycle (light time, 07:00 to 19:00), with food and water ad libitum unless otherwise specified. Ambient temperature (23° to 25°C) and humidity (55 to 62%) were automatically controlled.

### FISH and immunofluorescence staining

Mice were transcardially perfused with phosphate-buffered saline (PBS), followed by 4% paraformaldehyde. The brains were then postfixed in 4% paraformaldehyde at 4°C overnight and then placed in a 30% sucrose solution for 2 days. The brains were frozen in an optimal cutting temperature embedding medium, and 16-μm (for FISH) or 35-μm [for immunofluorescence (IF)] coronal sections were cut with a vibratome (Leica, no. CM3050 S). For FISH experiments, the slices were mounted on SuperFrost Plus slides and air dried. The multicolor FISH experiments were performed following the instructions of the RNAscope Fluorescent Multiplex Assay (ACD Bioscience). The probes used in this study include the following: Il1rapl2 (catalog no. 552341), Slc17a7 (catalog no. 501101), GAD1 (catalog no. 400951), Slc17a6 (catalog no. 428871), Etv1 (catalog no. 557891), Tshz2 (catalog no. 431061), Pou3f1 (catalog no. 436421), and Foxp2 (catalog no.428791). For IF, cryostat sections were collected and incubated overnight with blocking solution (1× PBS containing 5% goat serum, 5% bovine serum albumin, and 0.1% Triton X-100) and then incubated with the following primary antibody, diluted with blocking solution, for 1 day at 4°C: rabbit anti-c-Fos (1:2000; Synaptic Systems, no. 226003). Samples were then washed three times with washing buffer (1 × PBS containing 0.1% Tween-20) and incubated with the Alexa Fluor–conjugated secondary antibodies (1:500; Thermo Fisher Scientific, catalog nos. A11039, 21206, and 10042) for 2 hours at room temperature. The sections were mounted and imaged using a Zeiss LSM800 confocal microscope with EC Pan-Neofluar 10×/0.30 M27 or Plan-Apochromat 20×/0.8 M27 objectives or a Keyence microscope.

### Viral delivery

The following AAVs (with a titer of >10^12^) were purchased from Addgene: AAV1-Syn-Flex-jGCaMP7s (no. 104491), AAV5-hSyn-DIO-hM3D(Gq)-mCherry (no. 44361), AAV5-hSyn-DIO-hM4D(Gi)-mCherry (no. 44362), AAV5-hSyn-DIO-mCherry (no. 50459), AAV-Ef1α-DIO-hChR2(H134R)-EYFP-WPRE-pA (no. 20298), pAAV-Ef1α-DIO-eNpHR 3.0-EYFP (no. 26966), pAAV-hSyn-FLEx-mGFP-2A-synaptophysin-mRuby (no. 71760), and pAAV-hSyn-DIO-eGFP (no. 50457). The following viruses for monosynaptic retrograde tracing experiments were purchased from BrainVTA: rAAV-Ef1a-DIO-mCherry-F2A-TVA-WPRE-hGH-polyA (PT0023), rAAV-EF1a-DIO-RVG (PT0207), and RV-ENVA-ΔG-eGFP (R01001).

### Stereotaxic brain surgeries

The AAVs were injected through a pulled-glass pipette and the nanoliter injector (Drummond Scientific, 3-000-207, Nanoject III). The injection was performed using a small-animal stereotaxic instrument (David Kopf Instruments, model 940) under general anesthesia with isoflurane (0.8 liter/min; isoflurane concentration, 1.5%) in oxygen. A feedback heater was used to keep mice warm during surgeries. Mice were allowed to recover in a warm blanket before they were transferred to housing cages for 2 to 4 weeks before behavioral evaluation was performed. For chemogenetic activation or inhibition experiments, 0.1 to 0.15 μl of AAV5-hSyn-DIO-hM3Dq/hM4Di-mCherry was bilaterally delivered at a rate of 1 nl s^−1^ into the mPFC [AP: *+*1.94 mm, medial-lateral (ML): ±0.45 mm, and dorsal-ventral (DV): –2.4 mm] in *Il1rapl2*-Cre or *Foxp2*-Cre mice. For optogenetic experiments, following the viral injection of AAV5-EF1a-DIO-hChR2(H134R)/eNpHR3.0-EYFP into the mPFC, the fiber optic cannula (200 μm in diameter; Inper Inc.) was implanted 0.1 mm above downstream terminals in BLA (AP: –1.34, ML: ±2.9, and DV: –4.5 mm) or PT (AP: –0.46, ML: ±0.88, and DV: –3.68 mm), and was secured with dental cement (Parkell, no. S380) in *Il1rapl2*-Cre mice. For the fiber photometry experiment, the viruses of AAV1-Syn-Flex-jGCaMP7s were injected into the mPFC, and the fiber optic cannula was implanted 0.1 mm above the mPFC.

### Fiber photometry

The *Il1rapl2^+^* neuronal dynamics during social interaction were measured using fiber photometry. Mice were subjected to the behavioral test 3 weeks after virus injection and fiber implantation. For social interaction recording, in the habituation phase, mice were habituated to the fiber optic cord and an empty cage for 10 min in the home cage with normal bedding. In the training phase, stranger mouse 1 was put into the cage and was allowed to interact freely for 10 min. In the test phase, for the novel mouse interaction group, stranger mouse 2 was put into the cage, while for the familiar mouse interaction group, still stranger mouse 1 was put into the cage. In the test phase, behavioral events, such as start or end sniffing with an empty cage or a familiar or novel mouse, were identified manually and synchronized with fluorescence signal based on recorded videos by charge-coupled device cameras (SuperCircuits, Austin, TX). When GCaMP-expressing neurons were excited or inhibited, the GCaMP fluorochrome would be increased or decreased, and emission fluorescence was acquired and amplified with the RZ10X fiber photometry system. Then, the voltage signal data stream for 405 nm (as isosbestic control) and 465 nm (for GCaMP signal) was shown with Synapse software [Tucker-Davis Technologies (TDT)] and was exported, adapted from a previous study (*60*), filtered, and analyzed with MATLAB code provided by TDT offline data analysis tools (www.tdt.com/docs/sdk/offline-data-analysis/offline-data-matlab/fiber-photometry-epoch-averaging-example). The data were segmented on the basis of individual trials of different events. To calculate Δ*F*/*F*, a polynomial linear fitting was applied to the isosbestic signal to align it to the GCaMP7 signal, producing a fitted isosbestic signal that was used to normalize the GCaMP7 as follows: Δ*F*/*F* = (GCaMP7signal − fitted isosbestic)/fitted isosbestic signal. The *z*-score of Δ*F*/*F* of the heatmap was then calculated as

$$z-\text{score}=\frac{V_{\text{signal}}-\bar{V_{\text{basal}}}}{\text{SD}\left(V_{\text{basal}}\right)}$$

Data are presented using the mean and SD of the signal. Time 0 s indicates the starting time point of each event.

### Neuronal tracing

To identify where *Il1rapl2^+^*neurons form synapses, *Il1rapl2*-Cre mice were unilaterally injected with 0.1 to 0.15 μl of pAAV-hSyn-FLEx-mGFP-2A-synaptophysin-mRuby in the mPFC. Three weeks after the virus injection, the brain tissue was collected and processed for confocal imaging. For monosynaptic retrograde tracing, *Il1rapl2*-Cre mice were microinjected with 0.2 μl of viral cocktails (1:1) of AAV-EF1a-DIO-mCherry-TVA and AAV-EF1a-DIO-RVG into the mPFC, and 3 weeks later, the same location was microinjected with 0.2 μl of modified rabies virus RV-ENVA-ΔG-eGFP. To aid visualization, images were adjusted for brightness and contrast using ImageJ across the entire image.

### Cell counts

For the smFISH imaging, three consecutive brain slices from each mouse containing the mPFC region were imaged by confocal microscopy. The total number of cell bodies was visually identified and manually counted. Then, the average cell number for these three brain slices and overlap percentages of different fluorescence were calculated. The identification of cell bodies is based on fluorescence dots surrounding 4′,6-diamidino-2-phenylindole (DAPI)–labeled nuclei.

For the rabies virus tracing, labeled neurons in the mPFC (injection site), primary motor cortex, CLA, medial septal nucleus (MS), PIR, TH, and hippocampus (CA1) were counted either manually or with the automated cell counter function in ImageJ in every 30-μm section. Cell numbers of three biological replicates were averaged. The proportion of GFP*^+^* neurons in the individual brain region is calculated by dividing the total GFP*^+^* neurons of labeled brain regions. No statistical methods were used to predetermine sample sizes, but our sample sizes are similar to those used in previous studies (*61*).

### Behavioral assays

#### Three-chamber social interaction

Each chamber (30 cm by 30 cm by 30 cm) contains dividing walls with an open middle section to allow for access. Both outer chambers contain wire cups. Mice were given free access to the apparatus for 10 min (absent of other mice) to habituate and confirm initial unbiased preference. The time spent in each chamber was recorded, and the time spent in close interaction with the nose point within 2 cm of the enclosure (zone) was also recorded (EthoVision XT 14). To test for sociability, mice were placed into the middle chamber of the apparatus with one outer chamber containing one mouse (stranger 1) confined in a wired cup and the other chamber containing a Lego block. For social novelty preference, mice were again placed into the middle chamber with one chamber containing the familiar mouse (stranger 1) and the other containing the novel mouse (stranger 2) confined in wired cups. Male familiar and novel mice introduced for assay in social interactions matched the male test subject. For each phase, the test mice explored the entire arena throughout the 10-min trial. The time spent interacting with the empty wire, stranger 1, and stranger 2 mice during the 10-min session was recorded (*62*). The percentage of preference was calculated as follows

$$\begin{array}{c} \%\text{Preference to stranger}\;1/2=\\ \frac{\text{Time}\;\left(\;\text{stranger}\;1/2\;\text{zone}\right)}{\text{Time}\;\left(\text{stranger}\;1\;\text{zone}+\text{stranger}\;2\;\text{zone}\right)}\times 100 \end{array}$$

#### Novel object recognition

For habituation on day 1, mice were habituated in the experimental environment for 30 min in the absence of objects. During training on days 2 to 4, the mice were placed in the presence of two identical objects and allowed to freely explore for 10 min. For testing on day 5, one object was replaced with a novel object, and mice were allowed to freely explore for 10 min. The exploration times spent on each of the familiar (F) object and the new (N) object were recorded. The discrimination index (in percentage) was calculated as (N − F)/(N + F) × 100 for intergroup comparisons.

#### Fear conditioning test

The fear conditioning test was performed using the NIR Video Fear Conditioning Package for Mice (Med Associates, Vermont, USA). On day 1, after a 2-min habituation, the mice were exposed to electrical shock (0.5 mA for 2 s) by pairing five conditioned stimuli (CSs) (29 pips, 200 ms, 6-kHz pure tone, repeated at 1 Hz). The ITI (intertone interval) was 30 s. On day 2, the mice were placed in the same chamber as on day 1 for 5 min by pairing five CSs (ITI: 30 s) without electrical shock to assess cue-dependent fear conditioning. For evaluation of cue-dependent conditioning, the freezing scores were obtained by the VideoFreeze software system (Med Associates, Vermont, USA) and expressed as a percentage of the baseline activity.

### Optogenetic laser stimulation and chemogenic manipulation

Laser light was delivered through a 200-μm-diameter optic fiber connected to the laser (Shanghai Laser & Optics Century Co. Ltd., China, BL473T3-050 or YL589T3-050), which was controlled by a waveform generator (Keysight). For photostimulating ChR2, the blue light stimulation parameter is 10 to 15 mW at the tip of the fiber, 20 Hz, 10 ms per pulse for 5 minutes per phase. For NpHR photostimulation, a 532-nm laser (OEM Lasers/OptoEngine) generates constant light with a power of 8 to 10 mW at each fiber tip for 5 minutes per phase. For chemogenetic studies, all mice were intraperitoneally injected with CNO (2.0 mg kg^−1^ for the hM3Dq group and 5.0 mg kg^−1^ for the hM4Di group; Cayman Chemical Company, no. 16882) 30 min before the behavioral test.

### Statistics

All statistical analyses were performed using GraphPad Prism (version 9) software, and fiber photometry results were analyzed by MATLAB. All mice were randomly assigned to different groups, and data collection was randomized whenever possible. Mice that, after histological inspection, had the location of the viral injection (reporter protein), cannula implantation, or the optic fiber(s) outside the area of interest were excluded. Data collection and analysis were not performed blind to the conditions of the experiments. Most behavioral experiments were controlled by an automated computer system, and the data were collected and analyzed in an unbiased way.

One-way analysis of variance (ANOVA) followed by Tukey’s post hoc test analysis was used for multiple comparisons, Dunnett’s post hoc test analysis was used for multigroup comparison, and unpaired or paired Student’s *t* test was used for inter- and intragroup comparisons. No statistical methods were used to predetermine the sample sizes or to randomize the groups. A two-tailed *P* < 0.05 was considered statistically significant. For detailed statistical analysis, see the figure legend with each figure.
