# Supplementary material for: A molecularly defined mPFC-BLA circuit specifically regulates social novelty preference
Source: Sci Adv. 2025 Apr 23;11(17):eadt9008. doi: 10.1126/sciadv.adt9008 (PMC12017316; doi:10.1126/sciadv.adt9008)
Supplement: Supplementary file 1 — Figs. S1 to S9 [file sciadv.adt9008_sm.pdf]

Science Advances
AAAS

Supplementary Materials for

A molecularly defined mPFC-BLA circuit specifically regulates social novelty preference

Yiqiong Liu *et al.*

Corresponding author: Yi Zhang, yzhang@genetics.med.harvard.edu

Sci. Adv. **11**, eadt9008 (2025)
DOI: 10.1126/sciadv.adt9008

This PDF file includes:

Figs. S1 to S9

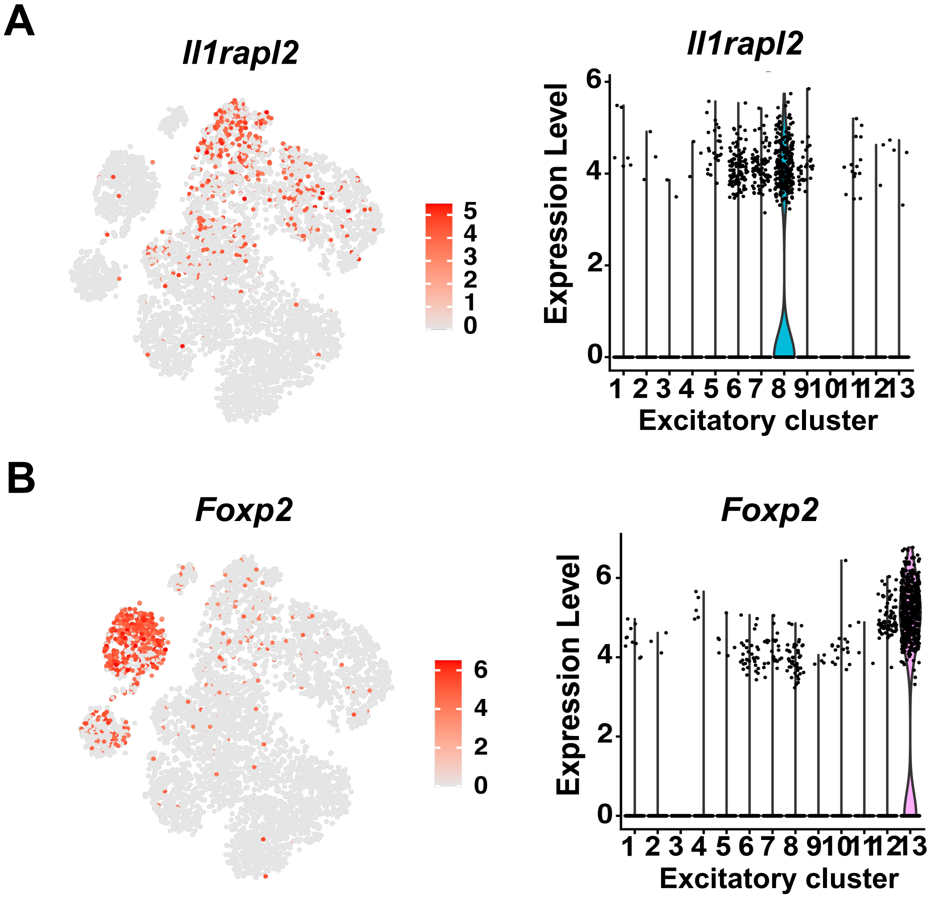

Fig. S1. Expression of the *Foxp2*⁺ and *Il1rapl2*⁺ neuron markers in the 13 mPFC neuron subtypes

A. Expression of *Il1rapl2*-specific marker was color-highlighted on broad t-SNE plot, and violin plot showing the expression of *Il1rapl2*-specific marker in the 13 mPFC excitatory neuron subtypes.

B. Expression of *Foxp2*-specific marker was color-highlighted on broad t-SNE plot, and violin plot showing the expression of *Foxp2*-specific marker in the 13 mPFC excitatory neuron subtypes.

Figure S2

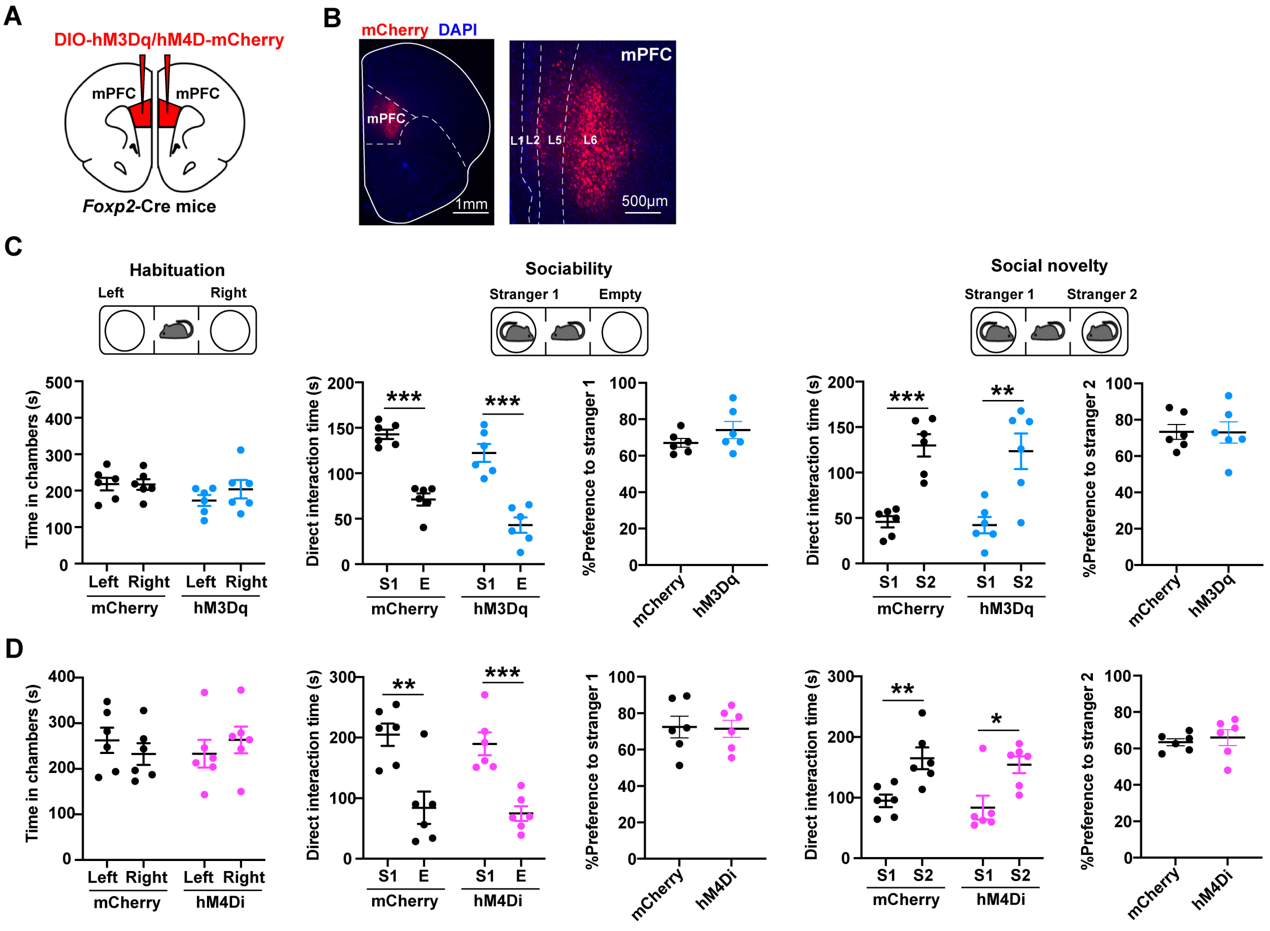

Fig. S2. mPFC *Foxp2*⁺ neurons are not involved in social behavior

A. Diagram of the injection site of AAV-DIO-hM3Dq/hM4Di-mCherry virus in mPFC of *Foxp2*-Cre mice.

B. Confirmation of mCherry expression in mPFC layer 6.

C. Three chamber interaction test in male *Foxp2*-Cre mice in both mCherry and hM3Dq groups in habituation stage (left), sociability stage (middle) and social novelty preference stage (right). E: empty, S1: Stranger 1, S2: Stranger 2. (***P*<0.01 and ****P*<0.001, student *t*-test).

D. Three chamber interaction test in male *Foxp2*-Cre mice in both mCherry and hM4Di groups in habituation stage (left), sociability stage (middle) and social novelty preference stage (right). E: empty, S1: Stranger 1, S2: Stranger 2. (***P*<0.01 and ****P*<0.001, student *t*-test).

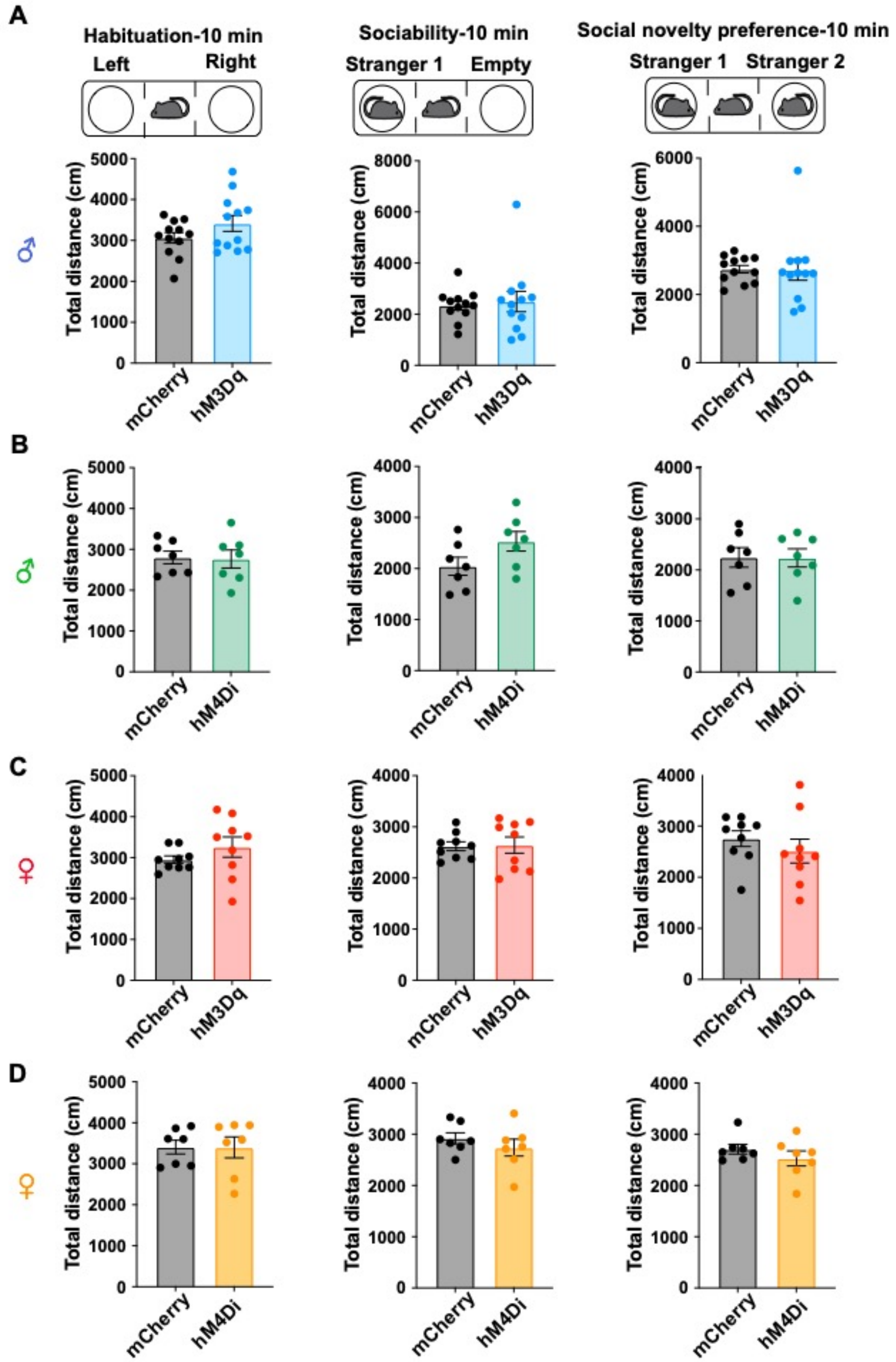

Fig. S3. Chemogenetic manipulation of the mPFC *Il1rapl2*$^{+}$ neurons does not affect locomotion during social behavior

A. Locomotion in three chamber interaction test in male *Il1rapl2*-Cre mice in both mCherry and hM3Dq groups in habituation stage (left), sociability stage (middle) and social novelty preference stage (right).

B. Locomotion in three chamber interaction test in male *Il1rapl2*-Cre mice in both mCherry and hM4Di groups in habituation stage (left), sociability stage (middle) and social novelty preference stage (right).

C. The same as panel A except that the test was performed using female mice.

D. The same as panel B except that the test was performed using female mice.

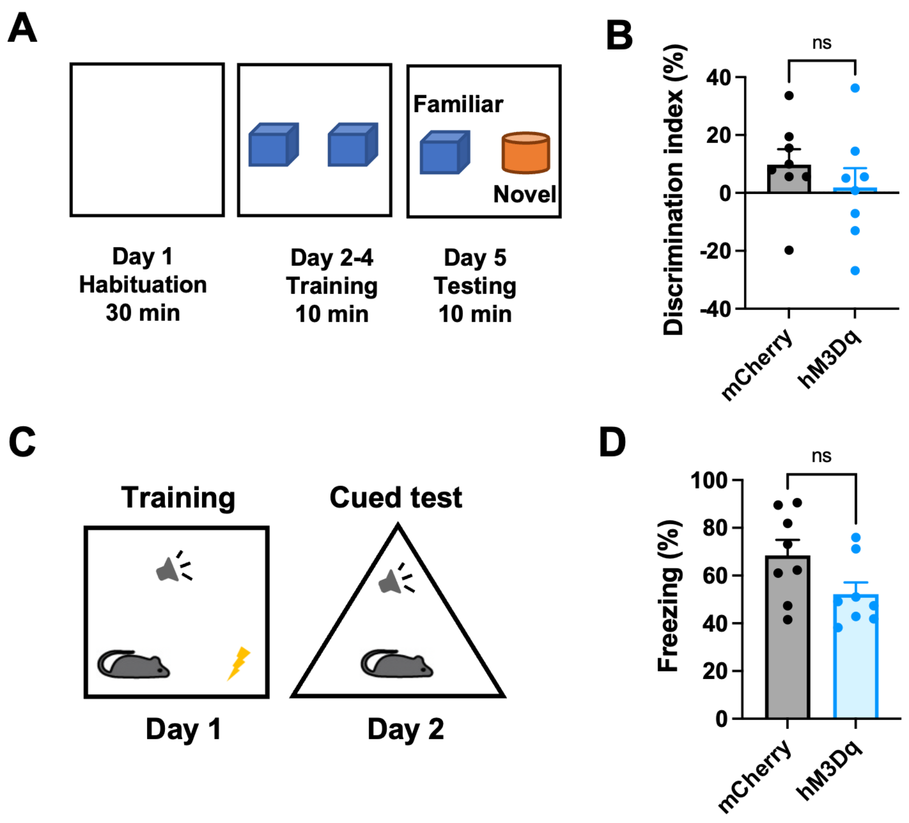

Fig. S4. mPFC *Il1rapl2*⁺ neurons are not associated with object or fear memory

A. Diagram for novel object memory test.

B. The discrimination index for novel object was calculated in both mCherry and hM3Dq groups in *Il1rapl2*-Cre mice.

C. Diagram for cued-fear memory test.

D. The percentage of freezing time was calculated in both mCherry and hM3Dq groups in *Il1rapl2*-Cre mice.

A

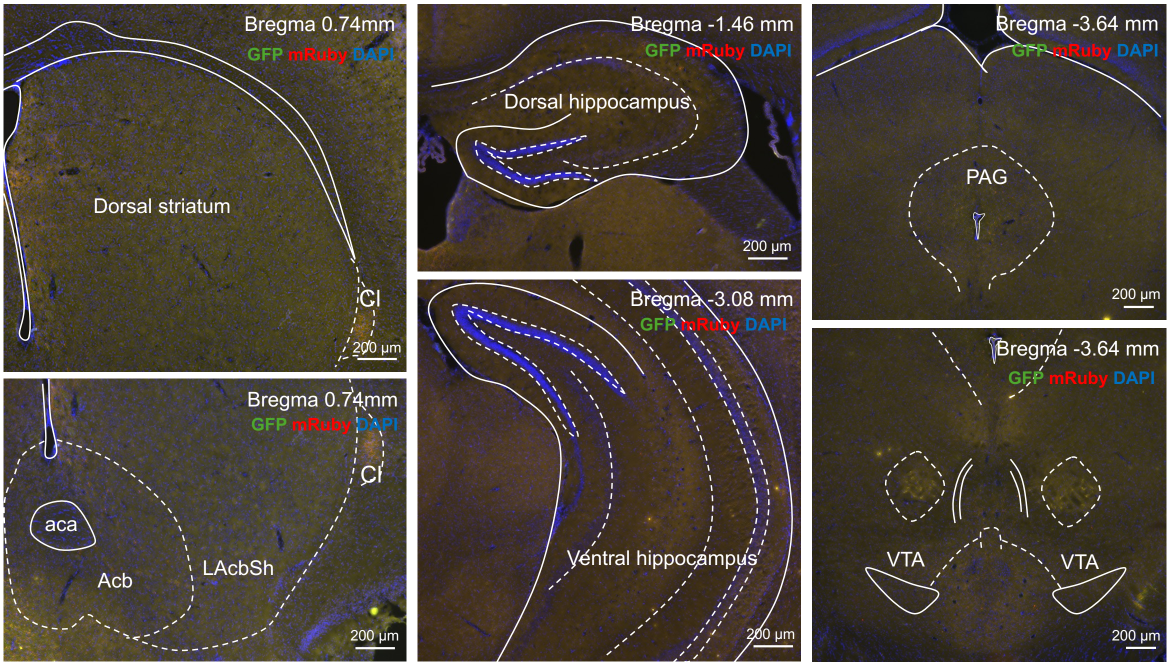

Fig. S5. Down-stream mapping of mPFC *Il1rapl2*⁺ neurons

A. Representative images showing brain regions without projection from mPFC *Il1rapl2*$^{+}$ neurons. Anterior commissure, anterior part (aca); accumbens nucleus (Acb); lateral accumbens shell (LAcbSh); periaqueductal gray (PAG); ventral tegmental area (VTA).

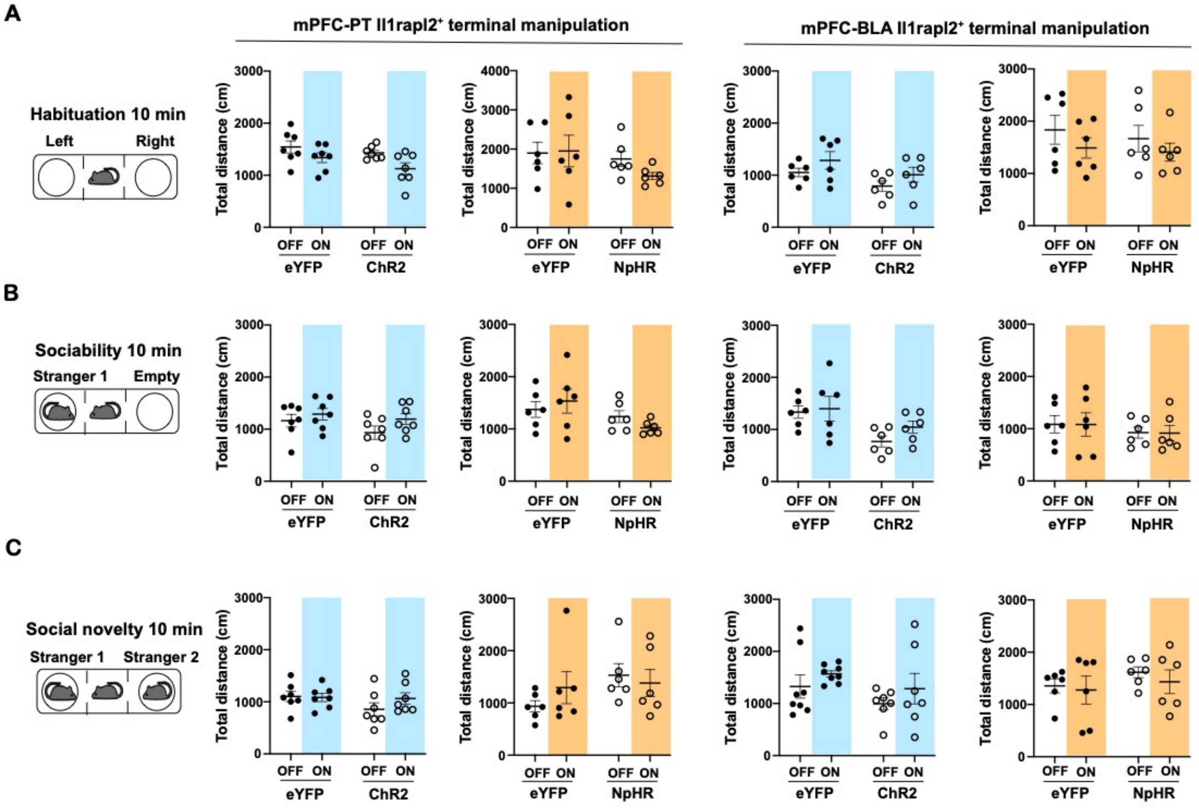

Fig. S6. Optogenetic manipulation of the mPFC *Il1rapl2*⁺ neuronal projections in PT or BLA does not affect locomotion during social behavior

A. Locomotion in three chamber interaction test during habituation stage in eYFP, ChR2 and NpHR groups in *Il1rapl2*-Cre mice with manipulation of mPFC-PT (left) or mPFC-BLA (right) *Il1rapl2*⁺ terminals.

B. Locomotion in three chamber interaction test during sociability stage in eYFP, ChR2 and NpHR groups in *Il1rapl2*-Cre mice with manipulation of mPFC-PT (left) or mPFC-BLA (right) *Il1rapl2*⁺ terminals.

C. Locomotion in three chamber interaction test during social novelty stage in eYFP, ChR2 and NpHR groups in *Il1rapl2*-Cre mice with manipulation of mPFC-PT (left) or mPFC-BLA (right) *Il1rapl2*⁺ terminals.

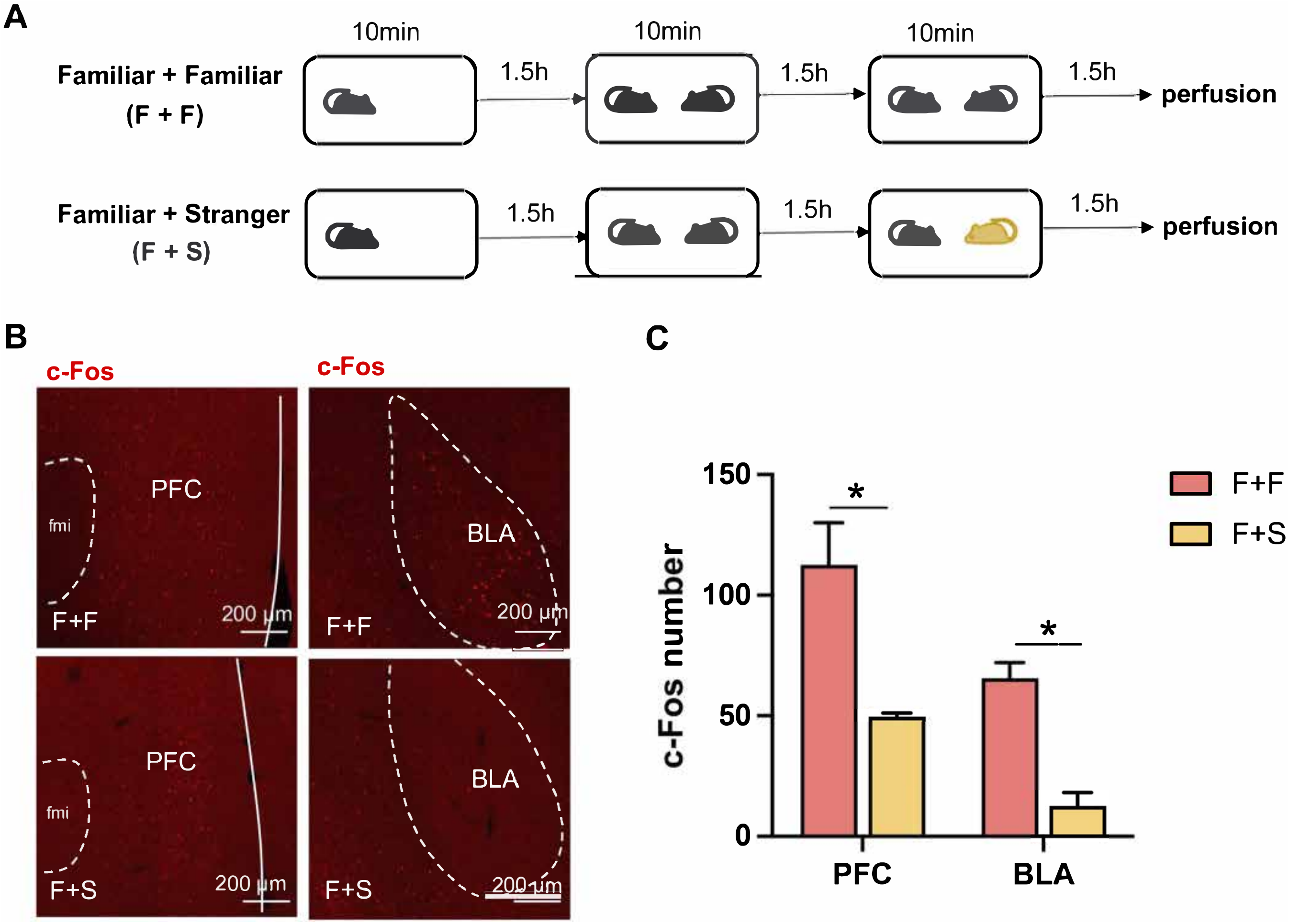

Fig. S7. cFos expression in different brain regions when facing familiar and novel mice

A. Schematic diagram for detecting cFos expression in different brain regions when facing familiar and novel mice.

B. Representative images of cFos expression in PFC and BLA when facing familiar and novel mice.

C. Quantification of the normalized cFos expression in PFC and BLA when facing familiar and novel mice. (*P<0.05, student t test).

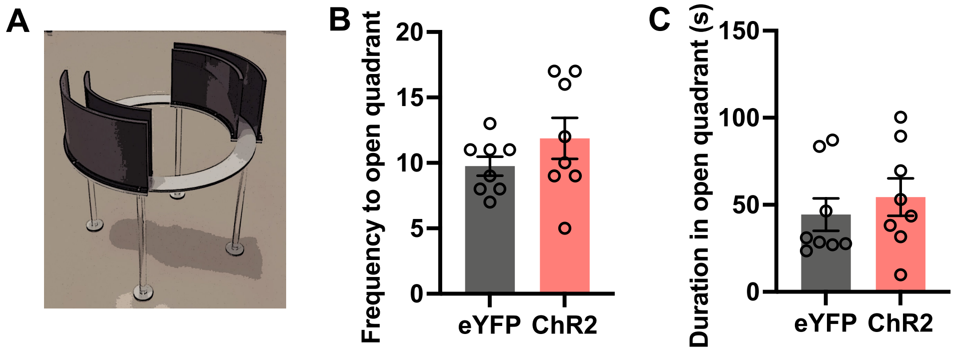

Fig. S8. Manipulation of the mPFC *Il1rapl2* -BLA projection does not affect anxiety.
A. Diagram for elevated zero maze.
B. The frequency to open arm was calculated in both eYFP and ChR2 groups in *Il1rapl2*-Cre mice.
C. The duration in open arm was calculated in both eYFP and ChR2 groups in *Il1rapl2*-Cre mice.

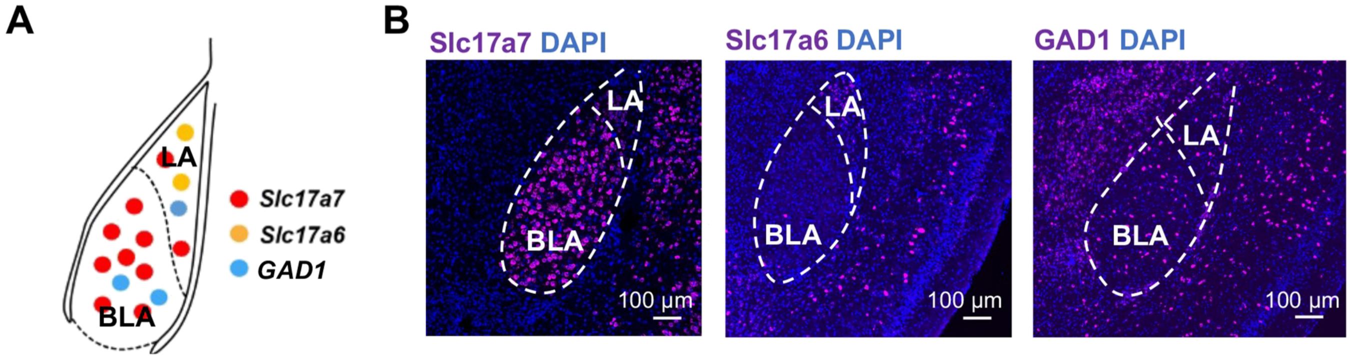

Fig. S9. Expression pattern of vGlut1, vGlut2 and GAD neurons in amygdala

A. Diagram illustrating the expression patterns of different neuron subtypes in amygdala, including vGlut1 (Slc17a7), vGlut2 (Slc17a6) and GAD.

B. RNA *in situ* florescence showing the expression pattern of vGlut1, vGlut2 and GAD neurons in amygdala.
